# Structural Characterization, Physicochemical Stability, and Antioxidant Activity of Rice Glutelin Hydrolysates

**DOI:** 10.3390/foods15152580

**Published:** 2026-07-23

**Authors:** Qi Zhang, Mengyan Jian, Yali Wang, Zimeng Wei, Yuehui Wang, Xiaoyu Bao, Wenping Ding

**Affiliations:** 1 College of Food Science and Engineering, Wuhan Polytechnic University, Wuhan 430023, China; qizhang21@163.com (Q.Z.); jianmengyan@163.com (M.J.); whyali181@163.com (Y.W.); 18674044803@163.com (Z.W.); xbao@whpu.edu.cn (X.B.); 2 Key Laboratory for Deep Processing of Major Grain and Oil, Ministry of Education, Hubei Key Laboratory for Processing and Transformation of Agricultural Products, Wuhan Polytechnic University, Wuhan 430023, China; whwangyh@163.com

**Keywords:** antioxidant, protein hydrolysates, rice glutelin, stability, Keap1-Nrf2

## Abstract

Rice glutelin hydrolysates (RGHs) with different degrees of hydrolysis (DH) were prepared using papain, and the structural characterization, physicochemical stability, and antioxidant activity of RGH were analyzed. Results showed that RGH primarily consisted of low molecular weight (MW) peptides (<3 kDa), with hydrophobic/aromatic amino acid content increasing with DH. Higher DH level led to reduced average particle size and zeta potential of RGH. Structurally, as DH increased, a decrease in α-helix content alongside increased β-sheet/random coil ratios was observed in RGH, indicating a transition towards a more disordered structure in RGH. Furthermore, the antioxidant activity of RGH was significantly enhanced with the increase in DH, with RGH-18 showing the highest bioactivity. RGH maintained stability and antioxidant capacity under gastrointestinal digestion as well as various environmental stresses, including varying pH and temperatures, and the presence of metal ions. Cellular experiments demonstrated that RGH-18 alleviated H_2_O_2_-induced oxidative damage in HepG2 cells, likely by inhibiting Keap1 and activating Nrf2 via the Keap1/Nrf2 pathway. This study supports the potential of RGH as a functional ingredient and provides insights for targeted rice peptide production.

## 1. Introduction

Environmental factors, aging populations, and modern fast-paced lifestyles have significantly raised the prevalence of non-communicable diseases, which are strongly linked to oxidative stress [1]. Oxidative stress refers to an imbalance between prooxidants and antioxidants [2]. An imbalance in the redox system results from the organism producing an excessive amount of free radicals or reactive oxygen species (ROS) when under oxidative stress. Excessive ROS can stimulate pathological redox signaling, resulting in cell damage and apoptosis, which can even increase the risk of various diseases [3]. Therefore, supplementing antioxidants is an important non-pharmacological strategy for preventing and inhibiting oxidative stress. Many studies confirmed the antioxidant effects of many nutrients and compounds such as phenols, flavonoids, peptides, etc., but their interactions with target genes and their impact on health are not yet clear [4].

Bioactive peptides are compounds composed of 2 to 20 amino acids that have biological activities, which are released during gastrointestinal digestion or processing. Compared with macromolecules, peptides are characterized by easy digestion and absorption, high bioavailability and safety. Plant- and animal-derived peptides are widely reported to inhibit oxidative stress via inhibiting ROS generation, enhancing antioxidant enzyme activities, eliminating free radicals, etc. [5]. Recently, numerous studies focused on the development of peptides with targeted regulatory effects, such as targeting the endogenous antioxidant defense Keap/Nrf2 signaling pathway [6,7]. For example, rice peptides AFDEGPWPK and SGDWSDIGGR can affect the Keap1-Nrf2 interaction via binding with Keap1 and thereby promote the expression of antioxidant-related enzymes [6]. Therefore, Nrf2, as a major redox regulator, can serve as a target of antioxidant peptides to improve cellular oxidative stress.

Rice protein, as an important source of plant-based protein, has the advantages of low allergenicity and reasonable amino acid composition, etc. Glutelin is the main storage protein of rice, accounting for about 66–78% of the total protein [8]. Compared to protein, rice peptides not only exhibit low molecular weight (MW) and good digestibility and absorption, but also exhibit multiple biological activities, including antioxidant and antihypertensive, and ACE-inhibitory activities [9]. The bioactivity of antioxidant peptides is related to their MW, amino acid composition, sequence, and spatial conformation. At present, most research focuses on the identification and activity validation of antioxidant peptides [10]; further research is needed on the enzymatic hydrolysis patterns and changes in peptide activity. Degrees of hydrolysis (DH) not only affect the structure of hydrolysates but also the composition, structure, and biological activity of peptides. Specific bioactive peptides or amino acids can be released from protein by using specific proteases and controlling the DH [11]. However, the effects of DH on the structural characterization, bioactivity and processing stability of protein hydrolysates are unclear, limiting the preparation and utilization of targeted antioxidant peptides in the food industry.

The current study investigated the structural properties and antioxidant activity of rice glutelin-derived peptides with different DHs. The structural characteristics, microstructure and antioxidant activities of rice glutelin hydrolysates (RGH) were investigated. The processing stability and gastrointestinal stability of RGH were also evaluated. Furthermore, the protective effects of RGH on the Keap1-Nrf2 signaling pathway in H_2_O_2_-damaged HepG2 cells were also explored. This study provides the potential relationship between the bioactivity and stability of rice peptides and DH, providing theoretical foundations for the precise preparation of antioxidant peptides.

## 2. Materials and Methods

### 2.1. Materials and Reagents

Rice (7% protein) was obtained from COFCO (Dongguan, China) Grain and Oil Industrial Co., Ltd. (Dongguan, China). Papain (100,000 U/mg) was obtained from Nanning Pangbo Biological Engineering Co., Ltd. (Nanning, China). Pepsin (P7000, ≥250 U/mg) and pancreatin from porcine pancreas (P7545, α-amylase ≥ 200 U/mg, protease ≥ 200 U/mg, and lipase ≥ 16 U/mg) were obtained from Sigma Chemical Co., Ltd. (St. Louis, MO, USA). O-Phthalaldehyde (OPA) was obtained from Shanghai Aladdin Biochemical Technology Co., Ltd. (Shanghai, China). Dithiothreitol (DTT) was obtained from Beyotime Biotechnology Institute (Shanghai, China). Antibodies for detecting Keap1, Nrf2, NQO1, and GAPDH were obtained from Proteintech (Chicago, IL, USA). Antibodies for detecting HO-1 were obtained from Servicebio Technology Co., Ltd. (Wuhan, China). Other reagents were of analytical grade.

### 2.2. Preparation of RGH

The preparation and modification of rice glutelin were determined according to our previous study [12]. The rice glutelin was dispersed in distilled water at a ratio of 1:10 (*w*/*v*), then the hydrolysis was conducted by 1.03% (E/S) papain under optimal conditions (pH 7.0, 50 °C). During the hydrolysis process, DH was measured by the OPA method [13]. The enzymatic reaction was maintained until reaching the specific DH (6%, 10%, 14%, and 18%). Subsequently, the hydrolysate solution was heated at 95 °C for 10 min to terminate the reaction; then, the solution after cooling was centrifuged at 8000× *g* at 4 °C for 20 min. Then, the collected supernatant was lyophilized to obtain RGH, and RGHs with different DHs were labeled as RGH-6, RGH-10, RGH-14, and RGH-18. All samples were stored at −20 °C for subsequent analysis.

### 2.3. Determination of MW Distribution

An HPLC system (Thermo Fisher, Waltham, MA, USA) coupled with a TSK G2000 SWXL column (7.8 × 300 mm, Tosoh, Tokyo, Japan) was employed to determine the MW distribution of hydrolysate. The mobile phase consisted of acetonitrile:water:0.1% TFA (20:80:0.1, *v*/*v*/*v*). A 10 μL volume of the samples was separated with a flow rate of 1 mL/min. The compounds were monitored at 220 nm using a UV detector. Standards included bovine serum albumin (66 kDa), cytochrome C (12.4 kDa), aprotinin (6.5 kDa), vitamin B12 (1.36 kDa), and reduced glutathione (0.307 kDa), and a calibration curve was constructed by plotting the retention time against the logarithm of molecular weight (R^2^ = 0.9982).

### 2.4. Amino Acid Composition Analysis

A total of 25 mg of sample was hydrolyzed with 3 mL 6 mol/L HCl in a sealed ampoule at 110 °C for 24 h. The hydrolysate was dried under nitrogen at 80 °C, reconstituted in a derivatization buffer, and filtered through a 0.45 μm membrane filter. For pre-column derivation, a 10 mL aliquot of the filtrate was reacted with 5 mL derivatization reagent (o-phthalaldehyde, OPA) at 60 °C for 1 h in the dark. After cooling and dilution, the solution was filtered through a 0.22 μm membrane filter and analyzed by HPLC (Thermo Fisher Scientific, 124 MA, USA) using an amino acid-specific ODS column (4.6 × 150 mm, Thermo Fisher Scientific, USA). Amino acids were identified by comparison of retention times with those of standard amino acids, and quantified using an external standard method with calibration curves constructed for each amino acid.

### 2.5. Determination of Average Particle Size and Zeta Potential

Zeta potential and particle size of the sample were detected using a zeta potential and nanoparticle size analyzer (BeNano180 ZetaPro, Dandong, China). The sample concentration was 1 mg/mL. The instrument parameters were configured as follows: a refractive index of 1.46 for the protein dispersed phase, 1.33 for the water dispersant, and an equilibration time of 2 min before measurement.

### 2.6. Circular Dichroism (CD) Spectroscopy

CD spectra were measured using a circular dichroism spectrometer (Jasco-J-1500, Tokyo, Japan). The sample concentration was 0.25 mg/mL, and the scanning rate was fixed at 100 nm/min. Spectra in the range of 190–260 nm were used to analyze its secondary structure.

### 2.7. Scanning Electron Microscopy (SEM) Analysis

The samples were observed using a SEM (SU8600, Hitachi, Tokyo, Japan). The particle morphology was observed at an accelerating voltage of 5.0 kV.

### 2.8. Intrinsic Fluorescence

The sample was dissolved in PBS at a concentration of 1 mg/mL for measurements. The sample was measured by a Fluorescence spectrophotometer (F-4600, Hitachi, Japan) at an excitation wavelength of 280 nm, with an emission wavelength in the range of 300–500 nm, with a slit width of 5 nm.

### 2.9. In Vitro Antioxidant Activity Assay

The ABTS radical scavenging activity, DPPH radical scavenging activity and ferric reducing antioxidant power (FRAP) were determined according to our previous study [12].

### 2.10. In Vitro Simulated Gastrointestinal Digestion

Rice glutelin was digested using the INFOGEST static simulated gastrointestinal digestion [14]. For gastric digestion, simulated gastric fluid (SGF) and CaCl_2_ (0.3 M) were mixed with the sample and adjusted to pH 3.0. The mixture was incubated at 37 °C for 2 h with pepsin (2000 U/mL). In the intestinal digestion phase, the pH was adjusted to 7.0, and SIF and CaCl_2_ were added, along with pancreatin (100 U/mL) and bile (10 mM). This mixture was incubated at 37 °C for 2 h before being heated to 90 °C for 10 min to inactivate the enzymes. All samples were lyophilized and stored at −20 °C for analysis.

### 2.11. Processing Stability Studies

The pH stability of hydrolysate (10 mg/mL) was also determined. The solutions were adjusted to pH levels of 2.0, 4.0, 6.0, 8.0, 10.0, and 12.0 using 1 M HCl or NaOH for 1 h, and antioxidant activity was assessed.

The heat stability of hydrolysate (10 mg/mL) was measured by incubation at 25 °C, 40 °C, 60 °C, 80 °C, and 100 °C for 1 h. After cooling to room temperature, the antioxidant activity of the sample was measured.

The ionic stability of hydrolysate (10 mg/mL) was determined by incubating it with NaCl at concentrations of 0, 0.1, 0.2, 0.5, 1, and 2 M and left at room temperature for 1 h before assessing antioxidant activity.

### 2.12. Cell Culture

HepG2 cells were maintained in MEM medium supplemented with 10% fetal bovine serum (FBS) and 1% penicillin–streptomycin solution. The cells were cultured in flasks under standard conditions (37 °C, 5% CO_2_, and saturated humidity).

### 2.13. Cell Viability

HepG2 cells were seeded in a 96-well plate at a density of 1 × 105 cells/mL. After 24 h, the medium was removed, and the cells were pretreated with fresh medium containing different concentrations of hydrolysate for 24 h. Then, the cells were treated with fresh medium with or without 500 µM H_2_O_2_. After 2 h, the CCK-8 assay was used to determine the cell viability at an absorbance of 450 nm.

### 2.14. Western Blot

Cells were collected and lysed using RIPA buffer supplemented with PMSF. The lysate was centrifuged (12,000× *g*, 5 min, 4 °C), and the supernatant was collected for protein quantification via a BCA assay. Equal amounts of protein were separated by SDS-PAGE and transferred onto a PVDF membrane. After blocking with 5% BSA in TBST for 1.5 h at room temperature, the membrane was incubated overnight at 4 °C with primary antibodies. Following three washes with TBST, the membrane was incubated with an HRP-conjugated secondary antibody for 1 h at 25 °C. Protein bands were visualized with an ECL detection kit, imaged using a Bio-Rad system, and quantified with ImageJ 2.14.0 (National Institutes of Health, Bethesda, MD, USA).

### 2.15. Statistical Analysis

Experimental data were expressed as mean ± SD. All experiments were conducted in triplicate. Statistical analysis was performed using SPSS 19.0 (SPSS Inc., Chicago, IL, USA) using analysis of variance and Duncan’s post hoc test. A value of *p* < 0.05 indicated statistical significance.

## 3. Results and Discussion

### 3.1. The MW Distribution of RGH

The MW distribution of RGH had a significant impact on its structure, physicochemical properties, and biological activity. As shown in Table 1, RGH consisted primarily of fractions below 3 kDa, representing 91.00–97.00% of the total content, confirming the dominance of low-MW peptides. Increasing DH led to the progressive transformation of high-MW peptides into low-MW fractions. Moreover, the peptide composition differed notably among the various DH levels. In the fractions with a 1–3 kDa range, RGH 6 and RGH 10 showed similar contents (approximately 30%), while RGH 14 and RGH 18 decreased significantly to 24.00–25.00% (*p* < 0.05). Notably, the proportion of peptides with MW < 1 kDa rose markedly with increasing DH, from 61.70% in RGH 6 to 73.22% in RGH 18 (*p* < 0.05), with no significant difference between RGH 14 and RGH 18. Our findings were consistent with research reported by Wu et al. [15], that enhanced hydrolysis promotes the conversion of high-MW peptides into low-MW peptides through increased peptide bond cleavage. Low-MW peptides generally exhibit stronger biological activity, enhanced absorption efficiency and high bioavailability compared to larger counterparts [9,16]. By donating electrons in free radical chain reactions, low-MW peptides enhance antioxidant activity, and they are more readily absorbed by small intestinal epithelial cells and transported to targeted organs. Therefore, RGH contained abundant small peptides with a promising potential for biological activity.

### 3.2. Amino Acid Composition of RGH

The amino acid composition and content of peptides were key determinants of their structural characteristics and physiological functions. As shown in Table 1, Glu, Arg, and Asp were the predominant amino acids of RGH, with contents of approximately 19 mg/100 g, 9 mg/100 g, and 10 mg/100 g, respectively. The content of essential amino acids ranged from 23.82 mg/100 g to 27.87 mg/100 g, accounting for 26.77% to 29.07% of the total amino acids, and Leu was notably abundant. According to earlier research, Leu residues positioned in non-terminal regions of peptides may increase their antioxidant ability [17]. Therefore, Leu may contribute to the biological activity of RGH. Additionally, RGH with various DHs contained hydrophobic amino acids ranging from 31.36 mg/100 g to 35.27 mg/100 g, while positively and negatively charged amino acids measured 17.40~18.15 mg/100 g and 28.41~29.16 mg/100 g, respectively. With no appreciable difference in the amounts of positively charged amino acids in the four RGHs, RGH-18 contained the highest levels of hydrophobic, charged, and aromatic amino acids, which may be associated with its highest potential antioxidant activity. By enhancing the membrane affinity through hydrophobic interactions and scavenging free radicals, the presence of hydrophobic amino acids may contribute to the antioxidant activity of peptides [18]. Aromatic amino acids are associated with enhanced antioxidant ability of peptides, with studies reporting that phenolic hydroxyl/hydrogen-bonding groups in Tyr and the indole ring of Trp serve as critical active sites [19]. Furthermore, charged amino acids may facilitate the absorption and transport of peptides in intestinal epithelial cells, which may enhance the bioavailability of antioxidant peptides [20].

### 3.3. Average Particle Size and Zeta Potential of RGH

**Figure 1:**
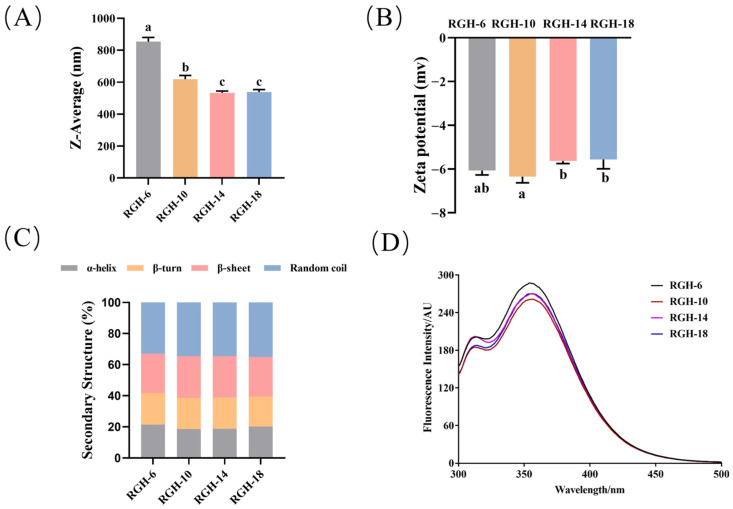
(**A**) Particle size, (**B**) zeta potential, (**C**) secondary structure, and (**D**) intrinsic fluorescence spectra of RGH under different DHs. Values are expressed as mean ± SD, *n* = 3. Bars labeled with different lowercase letters indicate significant differences between distinct treatments applied to the same hydrolysate, while uppercase letters indicate significant differences between different hydrolysates under identical treatment conditions, *p* < 0.05.

The average particle size and zeta potential of RGHs with different DHs are shown in Figure 1A,B, respectively. RGH-6 exhibited the largest average particle size of 853.77 ± 27.01 nm. It indicated that, at a low DH, RGH retained a considerable amount of long peptide segments. Consequently, hydrophobic regions were substantially exposed and led to aggregation via hydrophobic interactions [21]. As the DH increased, the particle size of RGH first gradually decreased, which is due to peptide chains being cleaved into small fragments, dispersing the hydrophobic regions and limited aggregation. The particle sizes of RGH-14 and RGH-18 eventually stabilized around 533.40–538.03 nm. It was attributed to deep hydrolysis generating a large number of small peptides (<1 kDa); their short hydrophobic segments tended to form dispersed structures. This observation aligned with the results of MW distribution; as the DH increased, the content of high-MW peptides gradually decreased and transformed into low-MW peptides. Specifically, RGH-14 and RGH-18 exhibited a significantly higher percentage of small peptides (<1 kDa) than RGH-6 and RGH-10.

As shown in Figure 1B, with the increase in DH, the zeta potential of RGH first increased slightly and then decreased significantly, which was consistent with the findings of Wang et al. [22]. The zeta potential of RGH-6 was −6.07 ± 0.20 mV, indicating a moderate level of surface negative charge. This could be attributed to the relatively long peptide chains and their tendency to undergo intermolecular entanglement, which buries charged groups and limits their exposure to solution. RGH-10, with a zeta potential of −6.35 ± 0.28 mV, exhibited the strongest negative charge. This likely resulted from further hydrolysis releasing low-MW peptide, promoting conformational changes and exposing more hydrophilic or charged groups [23]. In contrast, the absolute values of the zeta potential for RGH 14 and RGH 18 decreased markedly, which was consistent with their stabilized small particle sizes. This may be due to the plastein reaction occurring in peptides at a high DH, and some charged groups may be buried.

### 3.4. Secondary Structure of RGH

CD spectroscopy was widely employed to investigate the secondary structure and dynamics of proteins. The secondary structures of proteins mainly consisted of α-helices, β-sheets, β-turns, and random coils, which displayed distinct signals within the wavelength range of 190–260 nm. As shown in Figure 1C, RGH consisted mainly of random coils (34.26 ± 0.90)% and β-sheets (26.13 ± 0.81)%, followed by β-turns (19.88 ± 0.38)% and α-helices (19.73 ± 1.41)%. Our previous studies indicated that the secondary structure of native rice gluten was dominated by β-sheets, followed by β-turns, while α-helices and random coils accounted for a lower proportion [12]. Compared to rice gluten, the secondary structure of RGH changed markedly, particularly with a pronounced increased in the proportion of random coils. It suggested that enzymatic hydrolysis disrupted the compact native conformation of rice gluten, thereby exposing buried hydrophobic regions and promoting structural rearrangement into more random coils. As the DH increased, the α-helix content in RGH decreased slightly, while the proportions of β-sheets and random coils increased somewhat; β-turns, however, remained relatively stable. This observation implied that enzymatic hydrolysis may disrupt the hydrogen bonds that stabilized α-helical conformations in RGH. The relative stability of β-turns could be attributed to their compact structure stabilized by intramolecular hydrogen bonds. Furthermore, the frequent presence of Pro and Gly in β-turns further restricts enzyme cleavage [24].

### 3.5. Intrinsic Fluorescence Spectroscopy of RGH

Fluorescence spectroscopy served as a remarkable technique for elucidating the conformational changes in proteins. As shown in Figure 1D, the fluorescence intensity of RGH-6 was the highest, RGH-10 showed decreased fluorescence intensity as DH increased, and the maximum absorption peak underwent a red shift, which was consistent with the findings of a previous study [25]. This could be related to the enzymatic hydrolysis breaking peptide bonds, which initially exposed specific functional groups (e.g., hydrophobic residues). The exposure of these groups subsequently promoted intermolecular hydrophobic interactions and molecular rearrangement, leading to a more compact protein conformation. The concomitant masking of interior groups (such as tryptophan residues) resulted in a decrease in fluorescence intensity. However, with further hydrolysis, RGH-14 and RGH-18 exhibited increased fluorescence intensity. This phenomenon could be attributed to some peptides being further cleaved into amino acids during prolonged hydrolysis, which increased exposure of characteristic aromatic amino acids [26]. Furthermore, with decreasing hydrophobic groups, hydrophobic peptide segments might have degraded into small peptides or free amino acids, while free amino acids or peptides might have concurrently recombined into new peptides.

### 3.6. Microstructure of RGH

**Figure 2:**
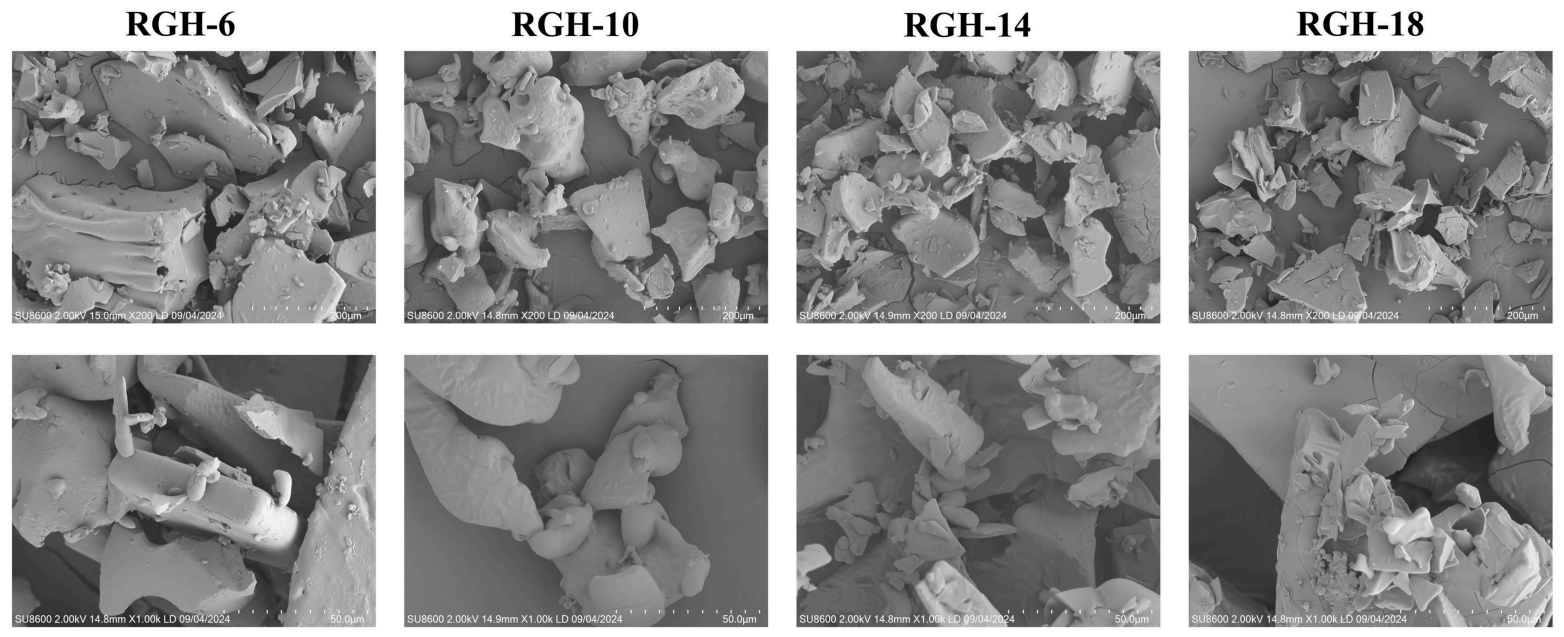
Scanning Electron Microscopy images of RGH at different DHs and magnifications (200× and 1000×). Scale bar 20 µm and 50 µm.

The microstructural characteristics of RGH were observed by SEM. As shown in Figure 2, RGH-6 exhibited a continuous lamellar structure with a relatively smooth surface and dense packing, with a few broad interlamellar pores, indicating localized enzymatic erosion at low DH. RGH-10 exhibited pronounced fracturing and fragmentation, with enlarged interlamellar pores, reduced particle size, and a loosely porous structure. It was likely caused by the selective cleavage of peptide bonds by the protease. RGH-14 exhibited a loose, honeycomb-like porous structure with uneven pores and smaller fragments. Notably, the roughened pore walls formed protrusions and interlaced microfibrillar structures. RGH-18 displayed uniformly dispersed nanoscale fragments with a loose porous surface, particularly revealing distinct honeycomb-like cavities under 1000× magnification. This could be attributed to the release of more small peptides at a high DH. Our findings were consistent with previous studies which have reported that high-degree hydrolysis degraded protein chains, increased specific surface area, and disrupted the protein structure. These structural changes resulted in loose conformations and extensive exposure of active sites, which are known to enhance functional properties [27,28].

### 3.7. In Vitro Antioxidant Activities of RGH

The antioxidant activities of RGH were evaluated by three methods. As evidenced by Table 2, with the increase in DH, the DPPH, and ABTS radical scavenging capacities, as well as the FRAP values of RGH, increased significantly. It was strongly associated with the high percentage of low-MW peptides in RGH, particularly those with MW below 3 kDa. Research has demonstrated that Glu and Asp contribute to the free radical scavenging ability of peptides, which was abundant in the RGH [29]. Furthermore, RGH was rich in hydrophobic and aromatic amino acids, which contributed to the antioxidant ability of hydrolysate. Notably, RGH-18 exhibited the strongest antioxidant activity among all hydrolysates, with its DPPH and ABTS radical scavenging capacity, and FRAP values increasing by 21%, 28%, and 58%, respectively, compared to RGH-6. These findings indicated that prolonged hydrolysis could enhance the antioxidant ability of RGH. Our findings aligned with previous research reporting a positive correlation between DH and the antioxidant ability of hydrolysates [28]. This positive correlation could be related to the high-DH-induced accumulation of low-MW peptides (<1 kDa) and enrichment of aromatic, charged, and hydrophobic amino acids in RGH-18. These structural features enhanced hydrogen-donating capacity and electron transfer efficiency through redox-active groups such as phenolic hydroxyls and amino groups [30].

### 3.8. In Vitro Antioxidant Activity of RGH During Gastrointestinal Digestion

**Figure 3:**
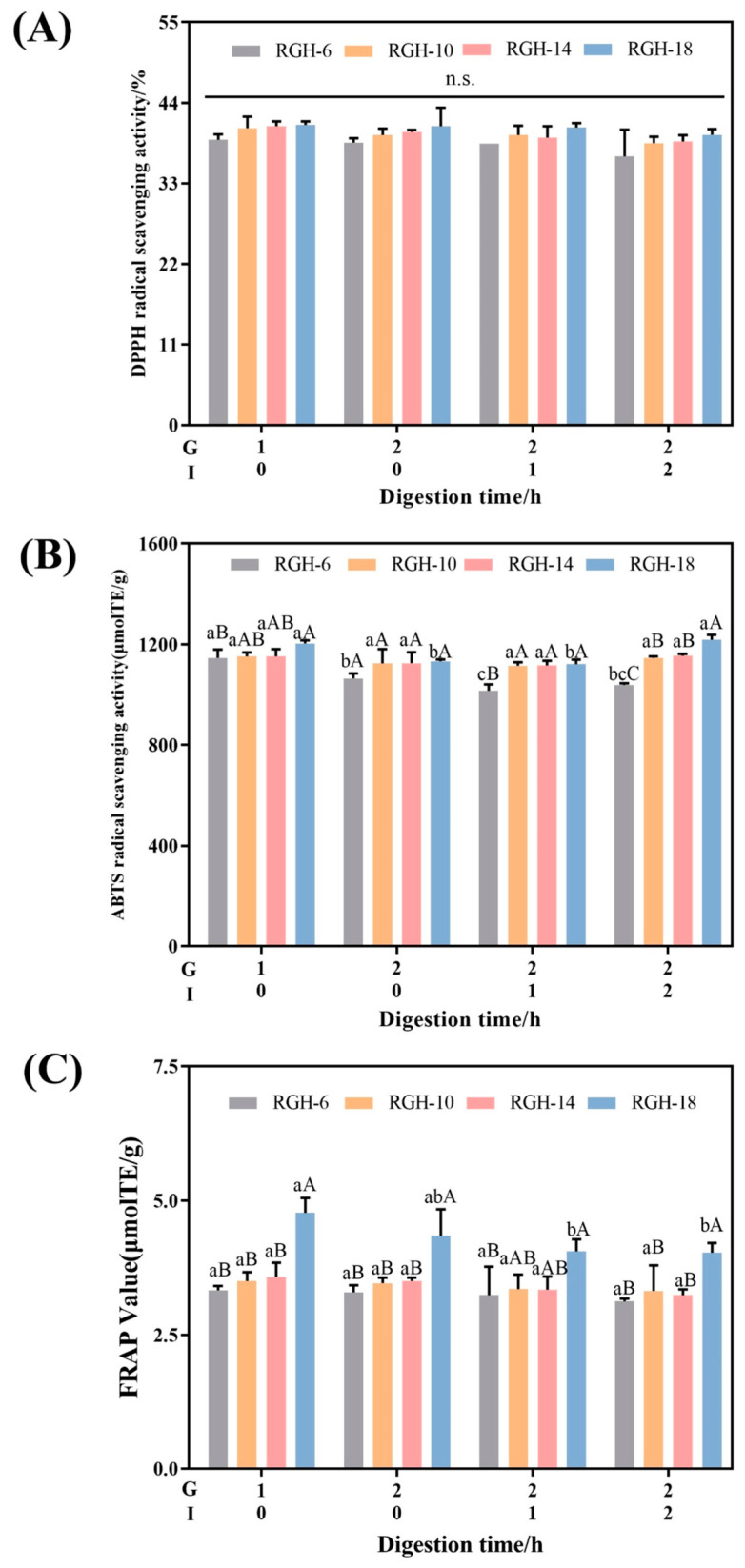
In vitro antioxidant activity of RGHs with different DHs during gastrointestinal digestion. (**A**) ABTS free radical scavenging activity; (**B**) DPPH free radical scavenging activity; (**C**) FRAP. Values are expressed as mean ± SD (*n* = 3). Bars labeled with lowercase letters indicate significant differences among hydrolysates at different digestion times within the same group; uppercase letters indicate significant differences between distinct hydrolysates at identical time points; “n.s.” indicates no significant difference, *p* < 0.05.

To exert their bioactivity, peptides must resist enzymatic degradation in the gastrointestinal tract, as digestive processes may diminish their bioactivity [31]. Therefore, the INFOGEST in vitro simulated gastrointestinal digestion model was employed to evaluate the digestive stability of RGH. As shown in Figure 3, throughout the entire simulated gastrointestinal digestion, the DPPH radical scavenging activities of all RGHs remained highly stable, with only slight fluctuations of approximately 2% observed across different time points and groups. However, regarding ABTS radical scavenging activity, distinct dynamic patterns were observed among the different RGH samples (Figure 3B). Specifically, RGH-6 exhibited a continuous decline, decreasing from 1146.31 ± 32.66 μmol TE/g to 1038.20 ± 7.59 μmol TE/g throughout the digestion, representing a reduction of approximately 9.4%. Conversely, RGH-10 and RGH-14 maintained consistent activity (Figure 3B). In contrast, RGH-18 displayed a unique “decrease–recovery” trend, where its ABTS value initially dropped from 1203.41 ± 12.62 μmol TE/g to 1120.80 ± 17.98 μmol TE/g, and subsequently recovered to 1217.98 ± 19.28 μmol TE/g during the intestinal phase. It was associated with the cleavage specificity of digestive enzymes. Pepsin preferentially hydrolyzes peptide bonds at hydrophobic residues (e.g., Phe and Leu), while trypsin has a wider range of cleavage sites and facilitates further degradation. Notably, RGH-18 demonstrated the highest ABTS radical scavenging activity throughout the digestion. The FRAP values of RGH-18 remained stable during gastric digestion but decreased significantly by approximately 15.5% in the intestinal phase, whereas the other RGH groups showed no significant changes. The FRAP values of RGH-18 remained stable during gastric digestion, but declined significantly in the intestinal phase, dropping from 4.35 ± 0.49 μmol TE/g to 4.06 ± 0.22 μmol TE/g, representing a reduction of approximately 6.7%, whereas the other RGH groups showed no significant changes. It could be attributed to abundant small peptides and fewer cleavage sites of RGH-18, as well as weaker hydrophobic interaction with enzymes. Evidence suggested that the majority of antioxidant peptides consistently exhibited poor stability during gastrointestinal digestion, showing decreased or even inactivated action; nonetheless, a few peptides were very slightly impacted [32]. This was possibly due to the breakdown of biopeptides by gastrointestinal digestive enzymes. RGH might contain a resistant bioactive peptide sequence or release novel antioxidant peptides upon digestion, which would necessitate further structural identification and peptide profiling in the future.

### 3.9. Analysis of the Processing Stability of RGH

#### 3.9.1. Effect of pH on the Antioxidant Activity of RGH

**Figure 4:**
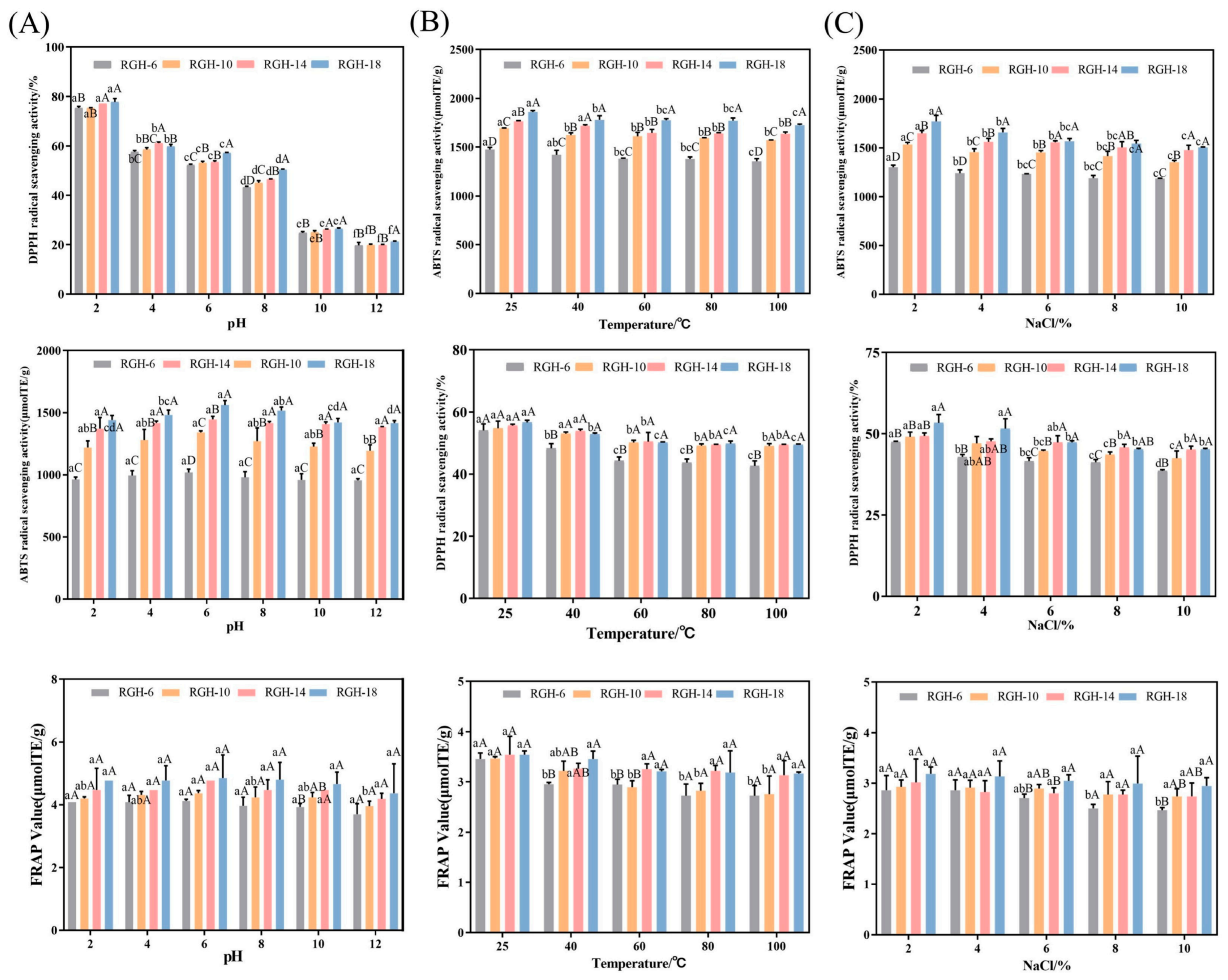
Effect of pH, temperature, and salt ions on the antioxidant activity of RGHs with different DHs. (**A**) pH stability; (**B**) heat stability; (**C**) ionic stability. Values are expressed as mean ± SD, *n* = 3. Bars labeled with different lowercase letters indicate significant differences between distinct treatments applied to the same hydrolysate, while uppercase letters indicate significant differences between different hydrolysates under identical treatment conditions, *p* < 0.05.

pH modulates both food processing efficiency and peptide bioactivity by altering the ionization states of key amino acid residues. Therefore, we further investigated the effect of pH on the stability of RGH. The DPPH radical scavenging rate of all RGH progressively decreased with increasing pH, as seen in Figure 4A, indicating that an acidic environment was favorable for the DPPH radical scavenging activity of RGH. The highest ABTS radical scavenging activity was detected by all RGH at a pH of 6, which decreased under strongly acidic and alkaline conditions. This could be attributed to the racemization and deamination reactions of peptides caused by strong acids and bases, affecting their electron transfer ability [33]. However, the FRAP values of all RGH remained relatively stable within the pH range of 2~12. While the majority of the antioxidant activity of RGH was still present, it was recommended to process, store, and use food in a neutral environment to keep its bioactivity.

#### 3.9.2. Effects of Temperature on Antioxidant Activity of RGH

The thermal stability of peptides is critical for their processing and manufacturing, as high temperatures may disrupt their secondary structure. As shown in Figure 4B, within 25–100 °C, the DPPH and ABTS radical scavenging capacities of all RGHs generally declined with increasing temperature, though high-DH RGHs retained relatively higher activity. FRAP assays further indicated that RGH-6 and RGH-10 exhibited poorer heat stability, with a marked drop-in antioxidant activity between 40 and 60 °C, while other RGHs remained more stable (Figure 4B). This thermal degradation trend may be explained by peptide denaturation or aggregation due to functional-group inactivation or chain cleavage at elevated temperatures [34]. Notably, RGH 18 consistently showed superior ABTS scavenging activity across all temperatures, suggesting enhanced thermal stability, which aligns with reported higher heat resistance of low-MW peptides (<1 kDa) [35]. Overall, RGH 14 and RGH 18 demonstrated greater heat resistance compared to low-DH RGHs.

#### 3.9.3. Effects of Salt Ions on Antioxidant Activity of RGH

This study investigated the effects of NaCl on the antioxidant activity of RGH. As shown in Figure 4C, the DPPH radical scavenging activity of RGH gradually decreased with increasing concentration of NaCl (2% to 10%). RGH-18 exhibited a DPPH radical scavenging rate of 53.88% at 2% NaCl, which decreased to 45.44% at 10% NaCl. This reduction could be attributed to the potential disruption of peptide spatial structures and amino acid side chain groups caused by the high concentration of NaCl, which may impair the antioxidant activity of RGH. In comparison, the ABTS radical scavenging activity of RGH exhibited a greater sensitivity to changes in salt ion concentrations, as illustrated in Figure 4B. High concentrations of NaCl may induce peptide flocculation or aggregation, potentially explaining the dose-dependent decline in radical scavenging activity observed with increasing salt concentration [36]. Consistently, Liu et al. [34] reported that 6–10% NaCl concentrations significantly decreased the ABTS radical scavenging activity of selenium-peptides. The FRAP values for RGH aligned with previous DPPH and ABTS radical scavenging activity. Except for RGH-6, which showed significant activity reduction, most RGHs maintained constant FRAP values at various NaCl concentrations. In contrast to other RGHs, RGH-18 exhibited superior antioxidant activity at various NaCl concentrations, indicating its remarkable resistance to ionic interference, and maintained antioxidant activity.

### 3.10. Protective Effects of RGH in H_2_O_2_-Damaged HepG2 Cells

**Figure 5:**
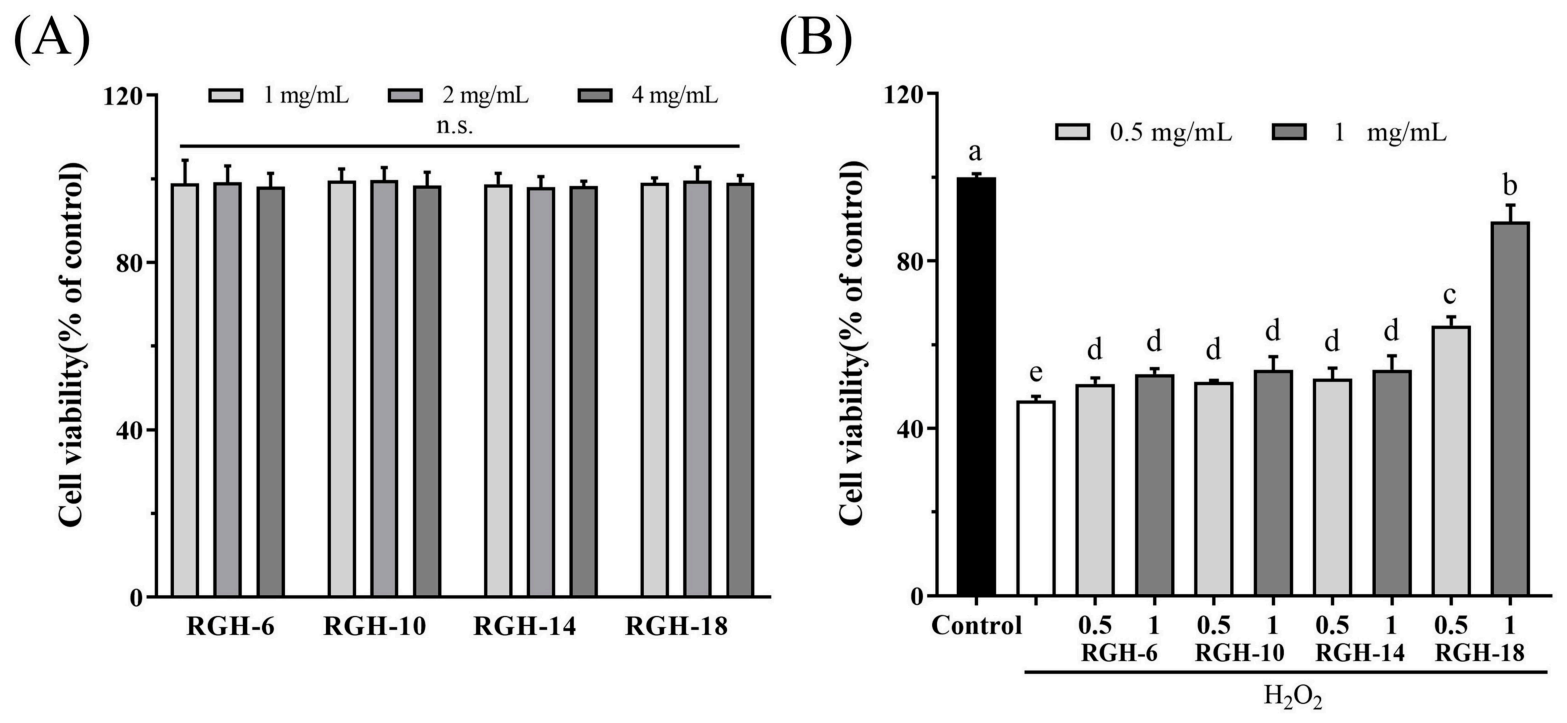
Effects of RGH on cell survival in H_2_O_2_-damaged HepG2 cells. HepG2 cells were pretreated with RGH at various concentrations before exposure to H_2_O_2_ (500 μM) for 2 h. (**A**) Cytotoxicity; (**B**) cell viability. Values are represented as mean ± SD, *n* = 6. Bars with different letters represent significant differences, *p* < 0.05.

An H_2_O_2_-damaged HepG2 cell model was constructed to assess the antioxidant protective effects of RGH at the cellular level. According to a preliminary experiment, after exposure to 500 μM H_2_O_2_, the cell viability of HepG2 cell was decreased to 46.67 ± 0.97%, which was used in this study. RGH showed no significant cytotoxicity at concentrations of 0.5–1 mg/mL (Figure 5A). Pretreatment with RGHs with different DHs for 24 h significantly alleviated the H_2_O_2_-induced decrease in cell viability (Figure 5B). Among them, RGH-18 exhibited the most potent protective effect in a dose-dependent manner, with cell viability recovered to 64.59 ± 2.07% and 89.39 ± 4.02% at 0.5 mg/mL and 1 mg/mL, respectively. It may be attributed to the high levels of hydrophobic amino acids in RGH, which enhanced peptide–lipid interactions, and aromatic amino acids that facilitated free radical scavenging and hydrogen atom donation. Moreover, abundant low-MW peptides also contributed to the antioxidant ability. Additionally, the peptide sequence and N-/C-terminal of peptides also had a major impact on antioxidant activity, which required further research.

### 3.11. Effects of RGH-18 on Keap1/Nrf2 Signaling Pathway

The Keap1/Nrf2 signaling pathway serves as a central endogenous antioxidant system, crucial for countering oxidative stress by upregulating protective enzymes [37]. Notably, food-derived bioactive peptides can activate this pathway by competitively binding to the active site of Keap1, thereby disrupting their interaction and stabilizing Nrf2 [38]. Therefore, we further assessed whether RGH could modulate the Nrf2-mediated pathway in cells.

**Figure 6:**
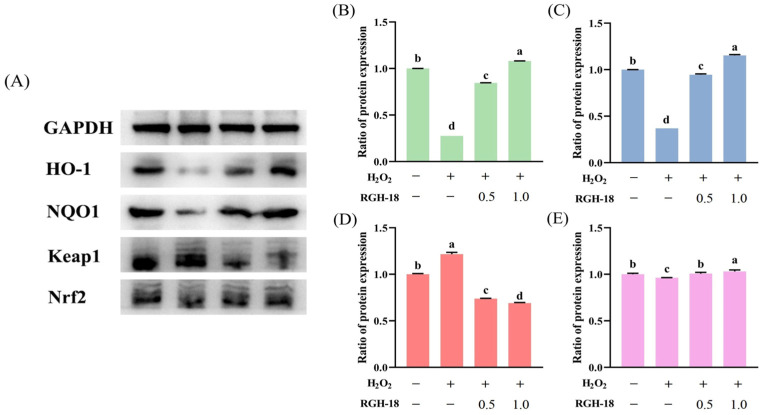
Effects of RGH-18 on Keap1/Nrf2 signaling pathway. (**A**) Western blot analysis of HO−1, NQO1, Keap1, and Nrf2; (**B**) densitometric analysis of protein expressions of HO-1, (**C**) densitometric analysis of protein expressions of NQO1, (**D**) densitometric analysis of protein expressions of Keap1, and (**E**) densitometric analysis of protein expressions of Nrf2. Values are represented as mean ± SD, *n* = 3. Bars with different letters represent significant differences, *p* < 0.05.

As shown in Figure 6, compared with the control group, H_2_O_2_-damaged cells significantly up-regulated the expression of Keap1 by 43.44%, and down-regulated the expression of Nrf2, antioxidant enzyme HO-1 and NQO1 by 22%, 46% and 38%, respectively. In contrast, treatment of RGH-18 reduced the expression level of Keap1, which was significantly lower than that of control group (*p* < 0.05), indicating a role of RGH-18 in suppressing Keap1. This reduction below the basal level may reflect a feedback regulatory mechanism, whereby robust Nrf2 activation downregulates Keap1 expression to sustain the antioxidant response [39]. Furthermore, incubation of RGH-18 led to a marked enhancement in the expression of Nrf2 and Nrf2-targeted genes HO-1 and NQO1, suggesting a pattern consistent with activation of the Nrf2-mediated signaling pathway. Moreover, at a concentration of 1 mg/mL, RGH-18 restored the decrease in HO-1 and NQO1 levels induced by H_2_O_2_, and its protein expression level was even higher than that of the control group. Nrf2 has been recognized as a modulator in inhibiting oxidative stress by activating the transcription and translation of endogenous antioxidant enzymes. Nrf2-targeted genes, HO-1 and NQO1, play a critical role in suppressing cell injury, apoptosis, and mitochondrial dysfunction [40]. A previous study reported that rice glutelin hydrolysate inhibited oxidative stress through regulating the Nrf2/Keap1 signaling pathway and activating the expression of Nrf2-targeted cytoprotective genes [41], which was consistent with our results. Rice peptide SGDWSDIGGR was reported to interact with the binding pocket of the Nrf2-binding site and modulate the Keap1/Nrf2 pathway to alleviate oxidative stress [10]. Collectively, these studies suggest that rice-derived peptide could reduce oxidative stress by regulating the Keap1/Nrf2 signaling pathway and serve as potential Nrf2/Keap1 regulators. However, the peptide profile of RGH-18 and its regulatory effect on key antioxidant peptides on the Nrf2/Keap1 pathway were still unclear and require further research.

## 4. Conclusions

RGHs with different DHs were prepared using papain, with over 90% of the peptides having an MW below 3 kDa. As DH increased, the content of low-MW peptides rose, accompanied by a significant decrease in average particle size and zeta potential. The α-helix content decreased, while the proportions of β-sheet and random coil structures slightly increased. The structure of RGH gradually loosened and disintegrated, with corresponding changes in its microstructure and secondary/tertiary conformation. RGH exhibited strong antioxidant activity, which was positively correlated with DH and attributed to the increased content of hydrophobic/aromatic amino acids and low-MW peptides. Further analysis demonstrated the high gastrointestinal digestive stability of RGH, along with retained strong antioxidant activity under acidic/alkaline conditions, temperature variations, and metal ion exposure. Moreover, RGH-18 enhanced cell viability and effectively alleviated oxidative stress by inhibiting Keap1 and activating Nrf2 along with its downstream cytoprotective genes. In summary, this study provides a foundation for the application of RGH as an antioxidant functional ingredient. Further research is needed to explore the bioactivity and bioavailability of key peptides.

## Figures and Tables

**Table 1:** Molecular weight distribution and amino acid composition of RGHs with different DHs.

Molecular Weight	RGH-6	RGH-10	RGH-14	RGH-18
	Molecular Weight Distribution (%)			
5–10 kDa	5.84 ± 0.19 ^a^	4.44 ± 0.56 ^b^	3.64 ± 0.25 ^c^	2.53 ± 0.09 ^d^
3–5 kDa	2.40 ± 0.24 ^a^	0.68 ± 0.16 ^b^	N.D.	N.D.
1–3 kDa	30.06 ± 0.89 ^a^	29.55 ± 2.68 ^a^	25.21 ± 0.74 ^b^	24.25 ± 0.55 ^b^
<1 kDa	61.70 ± 0.85 ^c^	65.33 ± 2.41 ^b^	71.15 ± 0.49 ^a^	73.22 ± 0.47 ^a^
Amino acid	Total amino acid content (g/100 g)			
Asp	8.56 ± 0.05 ^c^	8.88 ± 0.12 ^b^	9.07 ± 0.11 ^b^	9.71 ± 0.12 ^a^
Glu	19.85 ± 0.25 ^a^	19.92 ± 0.29 ^a^	19.46 ± 0.21 ^a^	19.45 ± 0.27 ^a^
Ser	4.54 ± 0.37 ^a^	5.15 ± 0.20 ^a^	5.17 ± 0.35 ^a^	5.02 ± 0.71 ^a^
Arg	9.42 ± 0.10 ^c^	10.01 ± 0.09 ^b^	10.43 ± 0.16 ^a^	10.53 ± 0.17 ^a^
Gly	4.45 ± 0.01 ^c^	4.50 ± 0.06 ^bc^	4.61 ± 0.06 ^b^	4.94 ± 0.08 ^a^
Thr *	2.81 ± 0.03 ^d^	2.99 ± 0.05 ^c^	3.12 ± 0.04 ^b^	3.32 ± 0.09 ^a^
Pro	3.79 ± 0.03 ^d^	4.04 ± 0.04 ^c^	4.24 ± 0.05 ^b^	4.55 ± 0.06 ^a^
Ala	4.76 ± 0.10 ^b^	4.69 ± 0.05 ^b^	4.76 ± 0.08 ^b^	5.01 ± 0.10 ^a^
Val *	4.66 ± 0.20 ^a^	4.01 ± 0.05 ^b^	3.90 ± 0.05 ^b^	3.93 ± 0.06 ^b^
Met *	1.09 ± 0.02 ^a^	1.19 ± 0.05 ^a^	1.19 ± 0.58 ^a^	1.05 ± 0.44 ^a^
Cys	0.36 ± 0.00 ^b^	0.44 ± 0.01 ^b^	0.73 ± 0.26 ^a^	0.58 ± 0.12 ^ab^
Ile *	2.90 ± 0.03 ^d^	3.11 ± 0.03 ^c^	3.30 ± 0.11 ^b^	3.71 ± 0.11 ^a^
Leu *	6.38 ± 0.06 ^c^	6.49 ± 0.07 ^c^	6.68 ± 0.11 ^b^	7.18 ± 0.10 ^a^
Phe *	3.50 ± 0.02 ^d^	3.96 ± 0.04 ^c^	4.25 ± 0.07 ^b^	4.74 ± 0.09 ^a^
His	3.74 ± 0.50 ^a^	3.35 ± 0.08 ^ab^	2.80 ± 0.16 ^c^	3.05 ± 0.08 ^bc^
Lys *	4.24 ± 0.03 ^b^	4.31 ± 0.09 ^b^	4.35 ± 0.07 ^b^	4.57 ± 0.16 ^a^
Tyr	3.92 ± 0.06 ^b^	4.06 ± 0.02 ^b^	4.43 ± 0.29 ^a^	4.51 ± 0.10 ^a^
hydrophobicity	31.36 ± 0.51 ^c^	32.01 ± 0.26 ^c^	33.47 ± 0.91 ^b^	35.27 ± 0.91 ^a^
positive electric charge	17.40 ± 0.54 ^b^	17.67 ± 0.10 ^ab^	17.59 ± 0.15 ^ab^	18.15 ± 0.37 ^a^
negative electric charge	28.41 ± 0.30 ^b^	28.80 ± 0.41 ^ab^	28.53 ± 0.24 ^b^	29.16 ± 0.19 ^a^
aromatic	7.42 ± 0.09 ^d^	8.02 ± 0.06 ^c^	8.67 ± 0.23 ^b^	9.25 ± 0.18 ^a^
Total	88.97 ± 0.57 ^c^	91.12 ± 1.05 ^b^	92.20 ± 0.57 ^b^	95.87 ± 0.66 ^a^

Note: Amino acids marked with * are essential amino acids. Hydrophobic amino acids: Ala, Cys, Val, Met, Ile, Leu, Tyr, Phe, and Pro; positively charged amino acids: His, Lys, and Arg; negatively charged amino acids: Asp and Glu; aromatic amino acids: Tyr and Phe. Different lowercase letters in the same column indicate significant differences (*p* < 0.05).

**Table 2:** In vitro antioxidant activities of RGHs with different DHs.

RGH	Antioxidant Activity		
	DPPH (%)	ABTS (μmolTE/g)	FRAP (μmolTE/g)
RGH-6	43.66 ± 2.61 ^c^	1352.76 ± 38.66 ^c^	7.84 ± 0.66 ^b^
RGH-10	47.44 ± 0.62 ^b^	1597.52 ± 2.94 ^b^	8.92 ± 0.75 ^b^
RGH-14	48.02 ± 2.17 ^b^	1631.50 ± 20.94 ^b^	9.98 ± 1.32 ^b^
RGH-18	52.82 ± 1.87 ^a^	1729.96 ± 34.64 ^a^	12.41 ± 1.80 ^a^

Note: Different lowercase letters in the same column indicate significant differences (*p* < 0.05).

## Data Availability

The original contributions presented in this study are included in the article. Further inquiries can be directed to the corresponding author.
